# High-density lipoprotein cholesterol as a therapeutic target for residual risk in patients with acute coronary syndrome

**DOI:** 10.1371/journal.pone.0200383

**Published:** 2018-07-11

**Authors:** Yuichi Ozaki, Atsushi Tanaka, Tsuyoshi Nishiguchi, Kenichi Komukai, Akira Taruya, Keisuke Satogami, Manabu Kashiwagi, Akio Kuroi, Yoshiki Matsuo, Yasushi Ino, Hironori Kitabata, Takashi Kubo, Takeshi Hozumi, Takashi Akasaka

**Affiliations:** 1 Department of Cardiovascular Medicine, Wakayama Medical University, Wakayama, Japan; 2 Department of Cardiovascular Medicine, Hidaka General Hospital, Gobo, Japan; Bascom Palmer Eye Institute, UNITED STATES

## Abstract

**Objective:**

The current guideline recommends lowering low-density lipoprotein cholesterol (LDL-C) for the primary management of dyslipidemia in patients at high-risk of cardiovascular events. Patients who have achieved LDL-C levels below the recommended targets may still experience cardiovascular events, suggesting additional therapeutic targets beyond LDL-C. The aim of this study was to investigate whether high-density lipoprotein cholesterol (HDL-C) levels had an impact on plaque stabilization in patients with acute coronary syndrome (ACS).

**Methods:**

This study consisted of 90 ACS patients with untreated dyslipidemia. In optical coherence tomography (OCT) analysis, a plaque with fibrous cap thickness ≦160 μm was defined as a high-risk plaque. We registered one high-risk plaque per one patient by baseline OCT imaging, and then administrated high-intensity statin. Based on the follow-up OCT results, patients whose registered plaque was no longer high-risk plaque were classified into a responder group and the remains into a non-responder group.

**Results:**

No differences were observed in the baseline LDL-C and HDL-C levels between the two groups. Reduction of LDL-C levels (δ LDL-C: −53 ± 21 mg/dL vs. −42 ± 29 mg/dL, *p* = 0.036) and increase of HDL-C levels (δ HDL-C: 2.5 ± 5.9 mg/dL vs. −0.3 ± 6.7 mg/dL, *p* = 0.039) were greater in the responder group. On multivariate logistic regression analysis, δ LDL-C levels (OR: 0.956, 95% CI: 0.921–0.993; *p* = 0.020) and δ HDL-C levels (OR: 1.143; 95% CI: 1.005–1.300, *p* = 0.041) were independent contributors for plaque stabilization.

**Conclusions:**

Increase of HDL-C levels is associated with plaque stabilization in patients with ACS. HDL-C could be a therapeutic target for residual risk management.

## Introduction

Today, atherosclerotic cardiovascular disease is the most important public health problem all over the world. Current guideline for the primary management of dyslipidemia in patients at high risk of cardiovascular disease recommends lowering low-density lipoprotein cholesterol (LDL-C) using statin or another anti-dyslipidemic agents [1]. High dose statin therapy has been provided lower LDL-C levels and plaque stabilization [2]. However, some patients could still suffer cardiovascular events, even though they have achieved LDL-C levels below the recommended targets. It should be a question to clarify the residual risk factors that affect the atherosclerotic progression of coronary plaque and result in cardiovascular events. This clarification must be suggesting additional therapeutic targets beyond LDL-C.

Intravascular optical coherence tomography (OCT) is a suitable method for plaque characterization that has provided a high-resolution imaging [3], and histological studies have demonstrated that OCT can identify the microstructure of atherosclerotic plaque, including fibrous cap thickness (FCT) and lipid core [4]. Our OCT study demonstrated that not only LDL-C levels but also high-density lipoprotein cholesterol (HDL-C) levels are associated with plaque vulnerability [5]. The aim of this study was to investigate the change of HDL-C levels had an impact on plaque stabilization using OCT in patients with acute coronary syndrome (ACS).

## Materials and methods

### Patient population

All 206 consecutive patients with successful percutaneous coronary intervention (PCI) for ACS (defined as ST-segment elevation acute myocardial infarction, non–ST-segment elevation myocardial infarction, or unstable angina) who underwent OCT for non-culprit lesion plaque, and untreated dyslipidemia (defined as serum LDL-C level >100 mg/dl) between March 2014 and February 2016, at Wakayama Medical University Hospital were included. Exclusion criteria were left main coronary artery disease, recommended coronary artery bypass grafting, cardiogenic shock, renal insufficiency with serum creatinine level >2.0 mg/dl, and current use of any lipid-lowering therapy according to self-reported or the previous and another hospital information as possible. This study was in compliance with the Declaration of Helsinki with regard to investigation in humans, and the protocol for this study was approved by the Ethics Committee of Wakayama Medical University (IRB #2293). We also obtained written informed consent from all the participants in this study for participation, medical procedure, and using of clinical data prior to coronary angiography.

### Study protocol

We performed an OCT examination in patients with successful PCI for ACS. The target plaque for this OCT examination was an intermediate non-culprit lesion plaque. We registered one high-risk plaque per one patient according to baseline OCT findings of the non-culprit plaques, and then administrated statin within 24 hours after PCI. OCT was performed at baseline and follow-up. Based on the follow-up (10.4 ± 2.1 months) OCT results, we defined the patients as a responder when the FCT became >160 μm, and the remains were as a non-responder. δ LDL-C and δ HDL-C levels mean that they were calculated by subtraction baseline from follow-up levels.

### OCT image acquisition and analysis

The OCT images were acquired using frequency-domain OCT (FD-OCT) as previously described [5]. The FD-OCT imaging system (C7-XRTM/ ILUMIEN OPTISTM, St. Jude Medical, St. Paul, Minnesota, USA) was used in the present study. Following a Z-offset adjustment, a FD-OCT image catheter (Dragonfly^TM^/ Dragonfly^TM^ JP/ ILUMIEN OPTIS^TM^ imaging catheter, St. Jude Medical, St. Paul, Minnesota, USA) was positioned so that its imaging lens was distal to the culprit lesion over a 0.014-inch conventional angioplasty guide-wire. All OCT images were obtained using an automatic pullback device traveling at a rate of 20 mm/s. To flush the coronary artery, contrast media at 37°C (Omnipaque^TM^ 350 Injection, Daiichi Pharmaceutical, Tokyo, Japan) was infused directly through the guiding catheter at a rate of 2.5 to 4.5 mL/s using an auto injector pump (Mark V; Medrad, Pennsylvania, USA). The FD-OCT images were digitally stored for offline analysis.

The OCT images were analyzed in a blinder fashion using a dedicated offline review system (St. Jude Medical, St. Paul, Minnesota, USA) at the core laboratory (Department of Cardiovascular Medicine, Wakayama Medical University, Wakayama, Japan). Serial OCT images at baseline and follow-up were reviewed side by side on the screen, and target plaques were matched based on the distance from landmarks like side branches and calcifications. OCT images were analyzed according to Consensus Standards for Acquisition, Measurement, and Reporting of Intravascular Optical Coherence Tomography Studies [6].

Fibrous cap was identified as a tissue layer, which is signal-rich homogenous region overlying a lipid core characterized by a diffusely bordered, signal-poor region on the OCT image. Lipid was semi-quantified according to the number of involved quadrants on the cross-sectional OCT image. Cap thickness was measured frame by frame and 3 times for each image to determine the thinnest site, and the average value was calculated. In this study, thin-cap fibroatheroma (TCFA) was defined as a plaque with a minimal FCT <65 μm. The lipid arc was measured on the frame with the largest lipid core by visual screening. Lipid length was calculated from the number of frames with lipid core. Fibrous or fibrocalcific plaques, which lack a fibrous cap and lipid core, were excluded from analysis. We compared minimum FCT at baseline with that of follow-up. In OCT analysis, a plaque with FCT less than 160μm was defined as a high-risk plaque, because our study previously demonstrated that plaque with FCT less than 160 μm was at risk for plaque disruption [7].

### Clinical parameters

The clinical parameters assessed were age, sex, and coronary risk factors, which consisted of hypertension (blood pressure ≥140/90 mmHg, and/or a history of taking antihypertensive medication), diabetes mellitus (fasting plasma glucose ≥126 mg/dl, casual plasma glucose ≥200mg/dl, or a diabetic pattern on 75-g oral glucose tolerance test), current smoking, and family history.

### Blood sampling and analysis

Blood samples were collected at baseline and follow-up in the fasting state. Serum samples were separated by centrifugation, stored at 4°C, and then analyzed (SRL Co., Ltd., Tokyo, Japan). Serum total cholesterol, HDL-C, triglyceride, and hemoglobin (HbA1c) levels were measured by enzymatic methods. Serum LDL-C level was calculated using the Friedwald equation. Serum levels of the inflammatory biomarkers high-sensitivity C-reactive protein (hs-CRP) were measured by a latex particle-enhanced turbidimetric immunoassay as reported previously [8].

### Statistical analysis

Variables were expressed as mean ± standard deviation, median [interquartile range], or counts (percentage). Comparisons between two groups were performed using Student’s *t*, Mann-Whitney *U*, or Chi-square test as appropriate. Multivariable logistic regression analysis was used to determine the contributors for plaque stabilization during follow-up. Age, male sex, coronary risk factors, δ LDL-C, δ HDL-C, and variables with a *p* value of <0.1 on the univariate assessment were entered into a multivariable model. Statistical analyses were performed using JMP version 12.2 (SAS Institute, Inc., Cary, North Carolina). A *p* value <0.05 was considered statistically significant.

## Results

### Patient population

**Fig 1:**
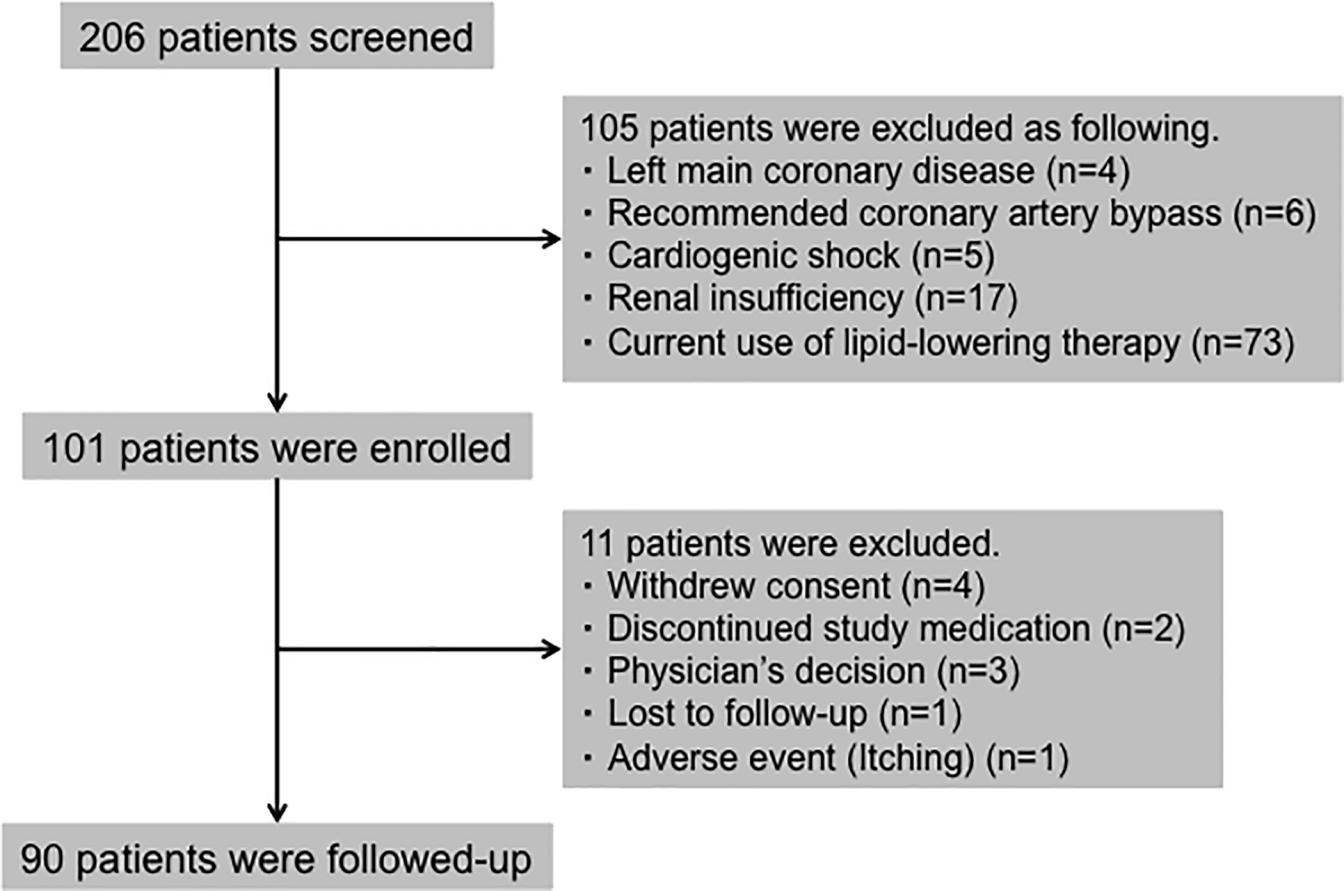
Study flowchart.

Among consecutive 206 patients, we excluded 105 patients who had a left main coronary artery disease (n = 4), recommended coronary artery bypass grafting (n = 6), cardiogenic shock (n = 5), and renal insufficiency (n = 17). We also excluded 73 patients caused by current use of lipid-lowering therapy, and then, we finally enrolled 101 patients. During the follow-up period, 11 patients were excluded due to withdraw consent (n = 4), discontinued study medication (n = 2), physician's decision (n = 3), lost to follow-up (n = 1), and adverse event (n = 1). Ultimately, we analyzed 90 patients for this study as shown in Fig 1.

### Patient characteristics and OCT findings

Forty-six patients (51%) were classified into a responder group according to the follow-up OCT findings. The baseline clinical characteristics of the study subjects are listed Table 1. There were no significant differences between the two groups. As a matter of course, no differences were observed in the baseline LDL-C and HDL-C levels. Table 2 shows the number of patients with statins and other lipid-modifying drugs at follow-up.

**Table 1:** Baseline characteristics.

	Responder	Non-responder	*p* value
Number, *n*	46	44	
Age (years)	65 ± 8	67 ± 10	0.29
Male gender	34 (74)	30 (68)	0.55
Coronary risk factors			
Hypertension	27 (59)	24 (55)	0.69
Diabetes mellitus	8 (17)	8 (18)	0.92
Current smoking	21 (46)	20 (46)	0.99
Family history	8 (17)	8 (18)	0.92
Total cholesterol (ml/dL)	201.3 ± 40.7	192.7 ± 24.5	0.22
Triglyceride (mg/dL)	125.4 ± 94.0	122.1 ± 76.7	0.86
LDL-C (mg/dL)	130.4 ± 27.9	123.6 ± 23.6	0.21
HDL-C (mg/dL)	42.8 ± 10.1	44.1 ± 10.2	0.56
HbA1c (%)	6.5 ± 1.8	6.4 ± 1.5	0.87
hs-CRP (mg/dL)	0.15 [0.07–0.46]	0.14 [0.08–0.51]	0.92
Medication			
Aspirin	3 (7)	6 (14)	0.31
ACEI or ARB	9 (20)	6 (14)	0.45
β blocker	8 (17)	4 (9)	0.25
CCB	13 (28)	11 (25)	0.73
Insulin	3 (7)	0 (0)	0.24
Target vessel			0.59
LAD	20 (43)	16 (36)	
LCX	11 (24)	9 (21)	
RCA	15 (33)	19 (43)	

Data are presented as mean ± standard deviation, median [interquartile range], or numbers (%).

ACEI, angiotensin converting enzyme inhibitor; ARB, angiotensin II receptor blocker; CCB, calcium channel blocker; HbA1c, hemoglobin A1c; HDL-C, high-density lipoprotein cholesterol; hs-CRP, high sensitivity C-reactive protein; LAD, left anterior descending artery; LCX, left circumflex artery; LDL-C, low-density lipoprotein cholesterol; RCA, right coronary artery.

**Table 2:** Lipid modifying drugs at follow-up.

	Responder (n = 46)	Non-responder (n = 44)	*p* value
Atorvastatin	21 (46)	33 (75)	0.01
Pitavastatin	25 (54)	11 (25)	0.01
Eicosapentaenoic acid	3 (7)	0 (0)	0.11
Ezetimibe	1 (2)	1 (2)	0.98

Data are presented as numbers (%).

There were no significant differences between the two groups in terms of baseline OCT findings of target plaque including FCT, lipid arch, plaque rupture, and intra-coronary thrombus (data not shown).

### Relationship between variation of lipid levels and plaque stabilization

**Fig 2:**
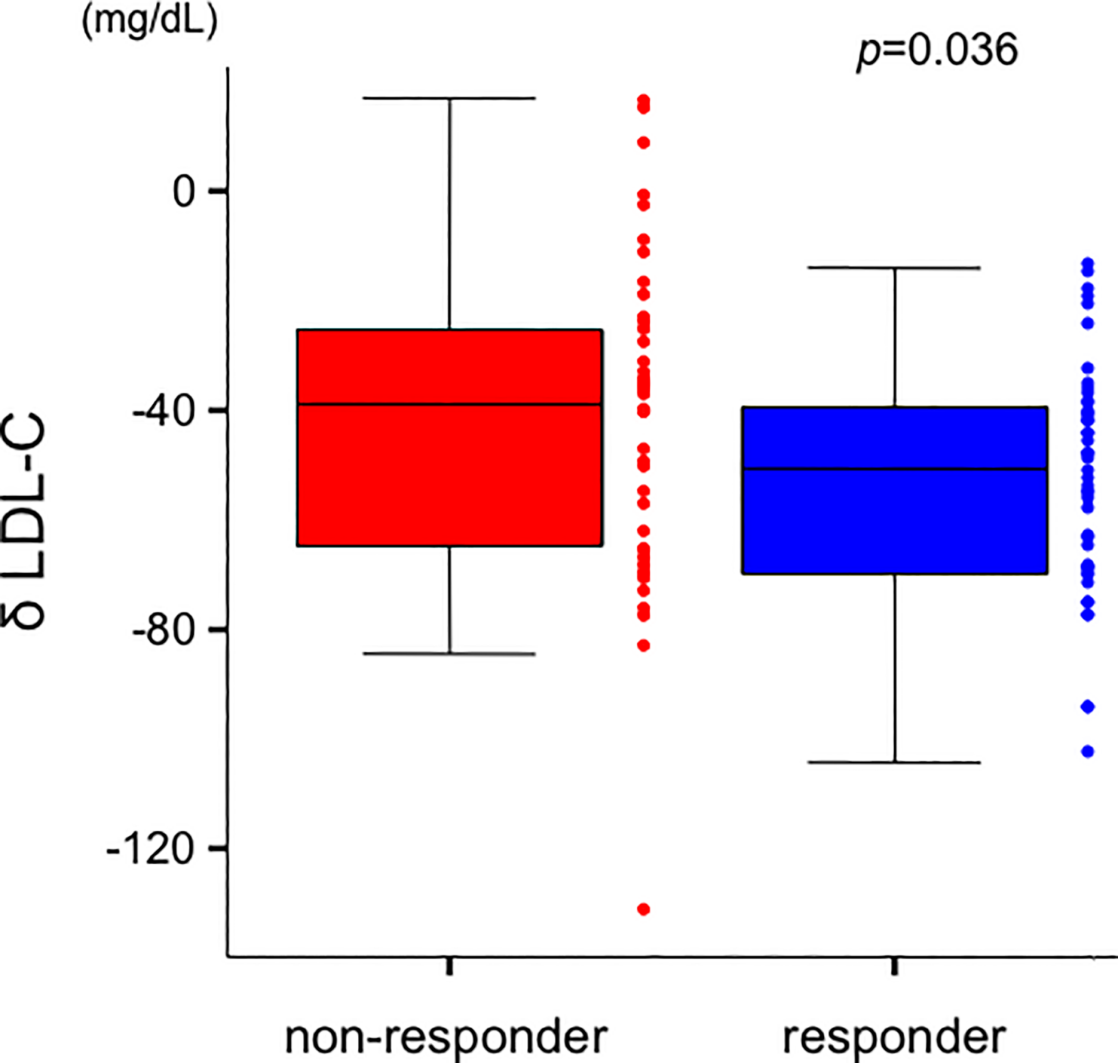
Comparison of δ LDL-C levels between the 2 groups. δ LDL-C levels in patients with responder group were greater than those in non-responder group (responder: −53 ± 21 mg/dL vs. non-responder: −42 ± 29 mg/dL, *p* = 0.036). Data are presented as box and whisker plots with median and 25th to 75th percentiles (boxes) and 10th to 90th percentiles (whiskers). LDL-C = low-density lipoprotein cholesterol.

**Fig 3:**
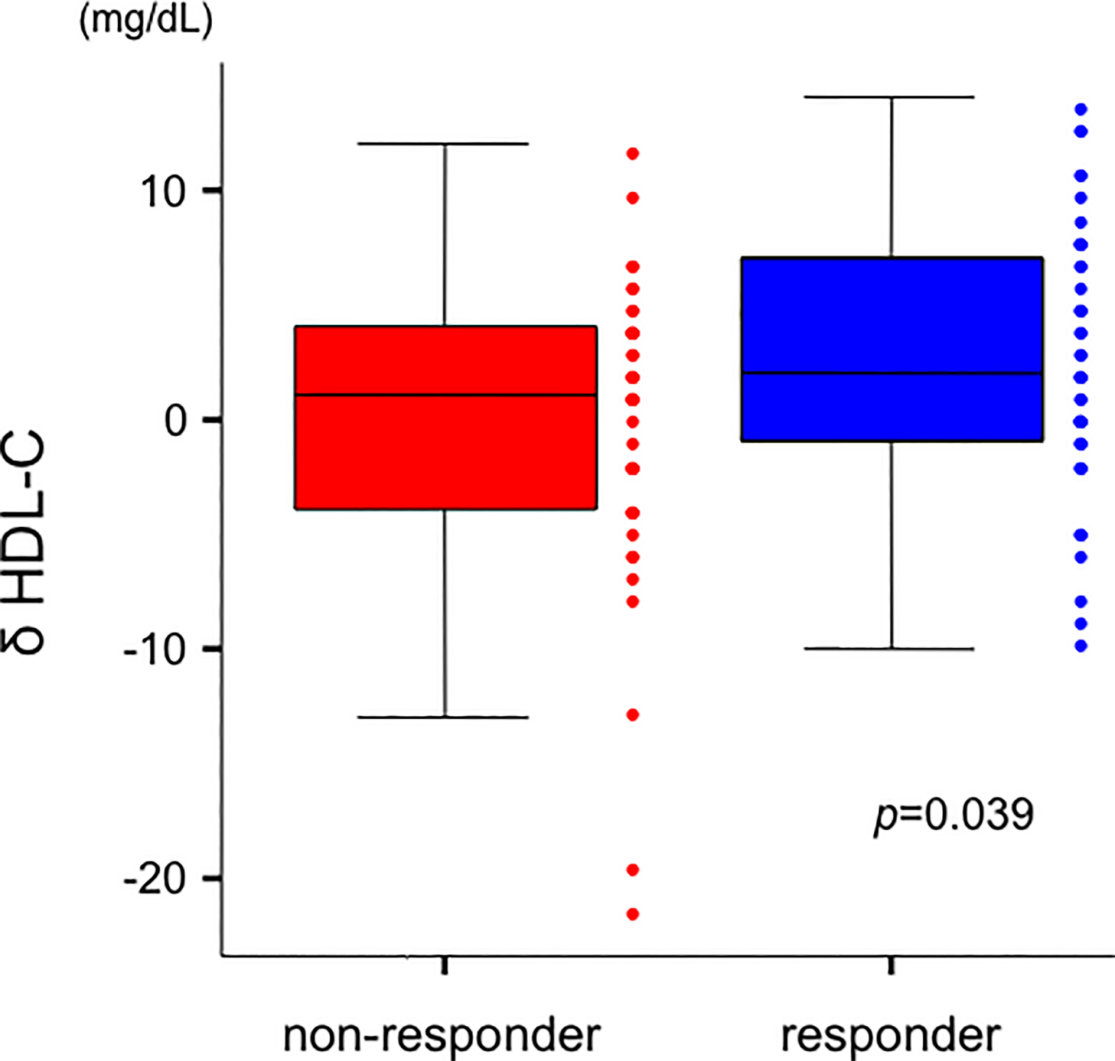
Comparison of δ HDL-C levels between the 2 groups. δ HDL-C levels in patients with responder group were greater than those in non-responder group (responder: 2.5 ± 5.9 mg/dL vs. non-responder: −0.3 ± 6.7 mg/dL, *p* = 0.039). Data are presented as box and whisker plots with median and 25th to 75th percentiles (boxes) and 10th to 90th percentiles (whiskers). HDL-C = high-density lipoprotein cholesterol.

No significant correlations were observed between the change in FCT and both δ LDL-C and δ HDL-C in overall patients (δ LDL-C; *r* = -0.176, *p* = 0.096, δ HDL-C; *r* = 0.107, *p* = 0.317, respectively). There was significant reduction in terms of LDL-C levels during the follow-up period in the responder group compared to those in the non-responder group (responder: −53 ± 21 mg/dL vs. non-responder: −42 ± 29 mg/dL, *p* = 0.036) (Fig 2). Furthermore, responder group indicated greater increase of HDL-C levels during the follow-up period than those in non-responder group (responder: 2.5 ± 5.9 mg/dL vs. non-responder: −0.3 ± 6.7 mg/dL, *p* = 0.039) (Fig 3).

### Predictive factors for plaque stabilization

Multivariate logistic regression analysis demonstrated that δ LDL-C (OR: 0.956, 95% CI: 0.921–0.993; *p* = 0.020) and δ HDL-C (OR: 1.143; 95% CI: 1.005–1.300, *p* = 0.041) were independent contributors for plaque stabilization. Moreover, no potential determinant for the change in FCT was observed analyzing with only baseline variables including age, gender, coronary risk factor, lipid profiles, and medications (data not shown).

## Discussion

To evaluate the association between HDL-C levels and plaque stabilization assessed by OCT is the focus of this research. In this study, we have demonstrated that not only lowering LDL-C but also raising HDL-C levels affected on plaque stabilization in patients with ACS.

### Lipid component and plaque morphology

It is well known that lowering LDL-C levels using anti-lipidemic agents such as statin lead to plaque stabilization. A number of clinical trials demonstrated that lowering LDL-C levels reduced major cardiovascular events [9–11]. Intravascular ultrasound (IVUS) studies revealed that statins suppress the progression of atherosclerosis or even enable regression of atheromatous plaque [12,13]. Recently, we revealed that significant association between lowering LDL-C levels using statin and plaque stabilization with the assessment of OCT findings such as FCT, lipid arch, or macrophage grade [8]. However, in spite of the statin therapy, there remains a residual risk even in patients with well-controlled LDL-C level. In the Framingham Heart Study, HDL-C levels had an inverse association with the incidence of coronary artery disease (CAD), that means HDL-C level was a more potent risk factor for CAD than LDL-C [14]. It was also reported that HDL-C level was a predictive factor of major cardiovascular events in patients treated with statin [15], even when LDL-C level was <70 mg/dl [16]. Previously, we demonstrated that HDL-C had an impact on fibrous cap thickening even after adjustment for LDL-C in patients with ACS using intra-coronary OCT imaging [5]. In the present study, we also shown that not only lowering LDL-C levels but also raising HDL-C levels had affected the plaque stabilization assessed by OCT in patients with ACS. These results may suggest that we should consider the additional therapy to reduce cardiovascular events.

Several studies have reported that low HDL-C levels are precious risk factor for CAD, and HDL-C potentially has various anti-atherogenic properties, including the regulation of reverse transport of cholesterol from cells from the arterial wall to the liver and steroidogenic organs [17–20]. The metabolism and a crucial anti-atherogenic function in HLD-C, that means reverse cholesterol transport, more especially, HDL-C-mediated efflux of cholesterol from non-hepatic cells and its subsequent delivery to the liver and steroidogenic organs, in which it is used for the composition of lipoprotein, vitamin D, bile acids, and steroid hormones [18–20]. It was reported that the ability of HDL-C to promote cholesterol efflux from macrophage foam cells was strongly and inversely associated with both subclinical atherosclerosis and obstructive CAD [21]. Take results into consideration in this study, a treatment strategy targeting LDL-C with statin may not be sufficient for CAD management. Therapeutic strategies of lipid treatment that involve additional targets beyond LDL-C reduction are required to improve clinical outcome and it is necessary to take more notice to modulation of HDL-C similar to that of LDL-C.

### Study limitations

First, the subjects in this study were administrated different statins including atorvastatin and pitavastatin, this may affect lipid profile or plaque stabilization. Second, some patients were administrated additional another anti-lipidemic agents such as eicosapentaenoic acid and ezetimibe. Third, the evaluation of plaque volume is not permitted due to the limited penetration depth of OCT. Therefore, it is difficult to estimate and quantify the amount of lipids on OCT, without IVUS. Fourth, we did not perform a 3-vessel assessment, therefore it is possible that some patients might have a vulnerable plaque in another coronary artery as previously reported [22]. Fifth, there were many potential confounders might effect on responder status that were not measured or consider in this analysis. Finally, since this is a single-center study involving a small sample, a study of larger patient populations from various centers with an independent core OCT laboratory is required to confirm the results, and our results may not be applicable to all patients with coronary artery disease because we excluded some patients with current use of anti-lipidemic treatments, plaques in the left main coronary artery, recommended coronary artery bypass grafting, cardiogenic shock, and renal insufficiency.

## Conclusions

As well as reduction of LDL-C levels, increase of HDL-C levels is associated with plaque stabilization assessed by OCT in patients with ACS. HDL-C could be a therapeutic target for residual risk management.

## Supporting information

S1 File All uploaded data. All data including age, gender, coronary risk factors, target vessels, laboratory data at baseline and follow-up, and medication were uploaded.
(XLSX)
